# Role of Cardiac Computed Tomography for Etiology Evaluation of Newly Diagnosed Heart Failure with Reduced Ejection Fraction

**DOI:** 10.3390/jcm9072270

**Published:** 2020-07-17

**Authors:** Dong Jin Im, Jong-Chan Youn, Hye-Jeong Lee, Kyungsun Nam, Young Joo Suh, Yoo Jin Hong, Jin Hur, Young Jin Kim, Byoung Wook Choi, Seok-Min Kang

**Affiliations:** 1 Department of Radiology, Research Institute of Radiological Science, Severance Hospital, Yonsei University College of Medicine, 50-1 Yonsei-ro, Seodaemun-gu, Seoul 03722, Korea; bluedjinn@yuhs.ac (D.J.I.); namks0216@yuhs.ac (K.N.); rongzu@yuhs.ac (Y.J.S.); uzzin@yuhs.ac (Y.J.H.); khuhz@yuhs.ac (J.H.); dryj@yuhs.ac (Y.J.K.); bchoi@yuhs.ac (B.W.C.); 2 Division of Cardiology, Department of Internal Medicine, Seoul St. Mary’s Hospital, College of Medicine, The Catholic University of Korea, 222 Banpo-daero, Seocho-gu, Seoul 16247, Korea; 3 Division of Cardiology, Severance Cardiovascular Hospital, Yonsei University College of Medicine, 50-1 Yonsei-ro, Seodaemun-gu, Seoul 03722, Korea; smkang@yuhs.ac

**Keywords:** heart failure with reduced ejection fraction, etiology, coronary computed tomographic angiography, delayed-enhanced cardiac computed tomography

## Abstract

Delayed-enhanced dual-energy computed tomography (DECT) can evaluate the extent and degree of myocardial fibrosis while coronary CT angiography (CCTA) is a widely accepted coronary artery evaluation method. We sought to describe the role of combined cardiac CT for the evaluation of underlying etiology in patients with newly diagnosed heart failure with reduced ejection fraction (HFrEF). Sixty-three consecutive patients (31 men, 63 ± 16 years) with newly diagnosed HFrEF were enrolled in this prospective study. Coronary artery disease and myocardial fibrosis were evaluated on CCTA and DECT, respectively, and the tentative underlying etiologies of heart failure (HF) were determined with combinations of findings from both CTs. Concordance between tentative etiologies from cardiac CT and final etiologies from clinical decisions within a 2-year follow-up was assessed. Eighteen patients were diagnosed with ischemic HF on initial cardiac CT, and the final diagnosis was not changed. Another 45 patients with nonischemic HF included tentative etiologies of dilated cardiomyopathy (*n* = 32, 71.1%), sarcoidosis or myocarditis (*n* = 8, 17.8%), amyloidosis (*n* = 2, 4.4%), noncompaction (*n* = 2, 4.4%) and arrhythmogenic right ventricular cardiomyopathy (*n* = 1, 2.2%). Five nonischemic HF patients showed different etiologies between initial cardiac CT and clinical decisions. The concordance between cardiac CT and clinical decisions was 92.1%. A high degree of concordance was achieved between tentative etiologies from cardiac CT and final diagnoses from clinical decisions. Combined cardiac CT is a feasible, safe and effective imaging tool for the initial evaluation of newly diagnosed HFrEF patients.

## 1. Introduction

Heart failure (HF) is a complex clinical condition that results from any structural or functional impairment of ventricular filling or blood ejection and has increased in prevalence with significant morbidity and mortality worldwide [1,2,3,4]. Early diagnosis and identification of the etiology behind HF are crucial because some etiologies indicate specific treatments [5,6,7,8]. 

In the past few years, multidetector computed tomography (CT) has been developed for cardiovascular imaging, especially for the noninvasive evaluation of coronary artery disease (CAD) [9]. Because approximately two-thirds of HF patients have ischemic etiology, identifying the underlying CAD is critical for HF management [10]. Therefore, cardiac CT can be considered a noninvasive imaging tool for assessing the likelihood of CAD in HF [1,11]. In addition, prior studies showed that cardiac CT can be used to evaluate myocardial fibrosis through myocardial delayed enhancement (MDE) imaging in both myocardial infarction and nonischemic cardiomyopathy with similar contrast kinetics for iodinated contrast agent and gadolinium [12,13,14,15]. Moreover, a recently developed dual-energy technique could strengthen the role of cardiac CT in MDE evaluation as it improved image quality with increased contrast-to-noise ratio through monochromatic imaging and iodine maps [16,17,18].

To determine the upstream pathophysiology and underlying etiology of newly diagnosed HF with reduced ejection fraction (HFrEF), appropriate evaluation for both coronary arteries and myocardial fibrosis is necessary [19]. Thus, we hypothesized that cardiac CT might be useful as a one-stop imaging tool as it allows coronary arteries and myocardial fibrosis to be examined simultaneously. However, there has been little research concerning this potential role of cardiac CT [20]. Therefore, we assessed the feasibility, safety and effectiveness of applying cardiac CT to the evaluation of unknown underlying etiology in newly diagnosed HFrEF through coronary CT angiography (CCTA) and delayed-enhanced dual-energy CT (DECT). We compared tentative etiologies of HF from cardiac CT and final etiologies of HF from two-year follow up clinical decisions.

## 2. Materials and Methods

### 2.1. Study Participants

From March 2014 to February 2015, we found 139 consecutive HF patients with the following inclusion criteria: adult patients (≥20 years of age) who were newly diagnosed with HF with relevant symptoms and signs, and with reduced left ventricular (LV) ejection fraction <40% on echocardiography [21]. We excluded 62 patients with a clinically presumed definite etiology of HF (47 patients for history of CAD, 8 for valvular heart disease, 3 for tachyarrhythmia, 2 for chemotherapy, and 2 for congenital heart disease). Ten patients with cardiogenic shock and/or acute coronary syndrome requiring urgent revascularization were excluded. Patients with decreased renal function of serum creatinine >1.5 mg/dL (2 patients), or iodine contrast allergy (2 patients) were also excluded. Finally, a total of 63 patients (31 men; mean age 62.6 ± 16.1 years, range 22–88 years, 47 inpatients and 16 outpatients) were enrolled. A flow diagram of enrolled study subjects and reasons for exclusion are summarized in Figure S1. All study protocols were performed following the relevant guidelines. The institutional review board and local ethics committee approved this prospective study and all included study participants gave informed consent (1-2014-0047).

### 2.2. Cardiac CT

**Figure 1:**
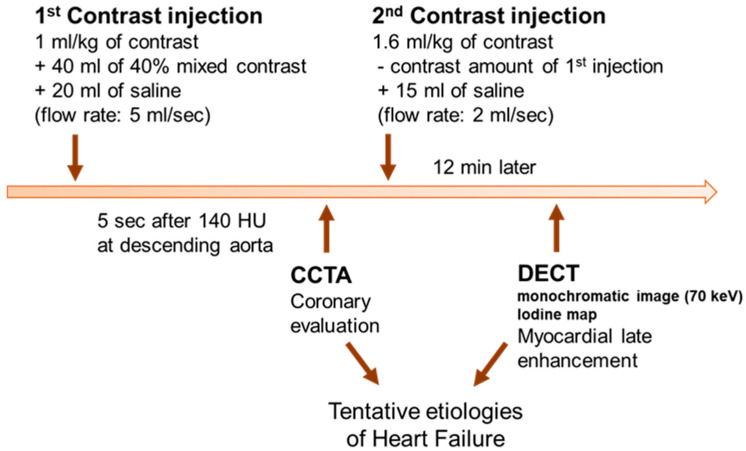
Flow diagram for cardiac CT scanning. CCTA = coronary computed tomographic angiography; DECT = dual-energy computed tomography.

Cardiac CT was performed with a second-generation dual-source CT (Somatom Definition Flash; Siemens Medical Solutions, Forchheim, Germany) within 3 or 4 days of initial HF diagnosis (Figure 1). An oral ß-blocker was administered to patients with heart rates ≥65 bpm and sublingual nitroglycerin was administered in all patients when there were no contraindications. For CCTA, a bolus of 1.0 mL/kg iopamidol (370 mg/mL of iodine, Iopamiro 370, Bracco, Italy) was injected into an antecubital vein at a flow rate of 5 mL/s followed by 40 mL of 40% blended iopamidol with saline and 20 mL of saline at 5 mL/s. The scan start was automatically initiated 5 s after reaching the threshold of 140 HU at the descending aorta. After the CCTA scan, iopamidol was additionally injected to reach the final total amount of 1.6 mL/kg in each patient followed by 20 mL of saline at 2 mL/sec. DECT was performed 12 min after the second injection. The scan range for the cardiac CT was from the carina to the diaphragm, and the field of view was adjusted according to heart size. CCTA was performed with the following parameters: prospective ECG-gated acquisitions at end-systole (heart rate > 65 bpm) or mid-diastole (heart rate ≤ 65 bpm), 120 reference kV and 250 reference mAs with Care kV and CAREDose4D (Siemens Healthcare, Germany), a 512 × 512 pixel matrix, a 64 × 0.6 mm slice collimation, and 0.33 sec rotation time. For CCTA, axial images were reconstructed using a slice thickness of 0.75 mm, an increment interval of 0.5 mm, and a medium-smooth convolution kernel of iterative reconstruction (I36f). Scanning parameters of DECT were as follows: retrospective ECG-gated acquisition with tube current modulation and ECG pulsing window in 60–80% of the R-R interval, 100 kV and 138 effective mAs for the A tube, 140 kV and 162 effective mAs for the B tube, a 512 × 512 pixel matrix, a 64 × 0.6 mm slice collimation and 0.33 sec rotation time. For DECT, the axial images were reconstructed at the mid-diastolic phase using a 0.75 mm slice thickness, a 0.5 mm increment interval, and a medium-smooth convolution kernel (D30f) at each tube voltage. CCTA images were transferred to an off-line workstation (Aquaris 4.4.11, TeraRecon, San Francisco, CA) and images were reformatted to the vertical and horizontal long axes, short axis, and curved multiplanar and volume rendering images, in addition to the axial images. Axial images at each tube voltage from DECT were transferred to a commercially available workstation (Syngo MMWP VE23A, Siemens Healthcare, Forchheim, Germany). For MDE, 70-keV monochromatic images and gray-scale iodine maps were created to the vertical long axis, horizontal long axis and short-axis planes with 8-mm slice thickness and no gap on the workstation. 

We prospectively evaluated the safety of combined CT in both inpatients and outpatients including the radiocontrast dye allergic reaction and contrast-induced acute kidney injury (CI-AKI). CI-AKI was defined as an absolute increase in serum creatinine levels by ≥0.5 mg/dL or a relative increase in serum creatinine by ≥50% from baseline observed within 72 h after contrast exposure. All enrolled patients underwent blood analysis for renal function at 72 h after combined cardiac CT imaging.

### 2.3. Image Analysis 

**Figure 2:**
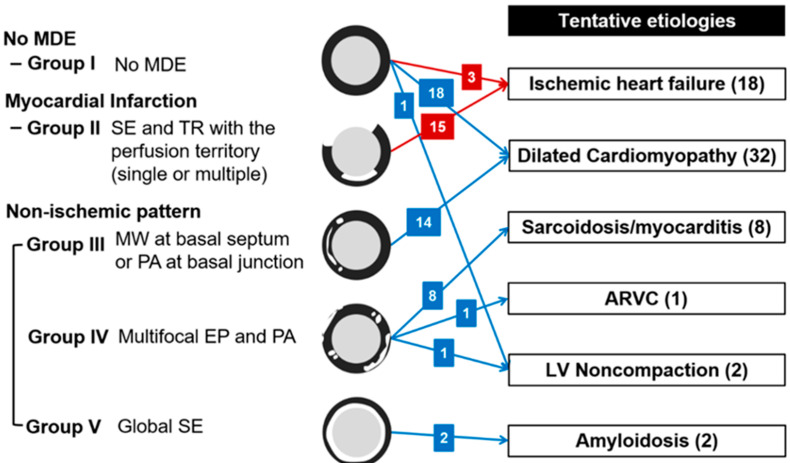
Tentative etiologies of heart failure from cardiac CT findings. Numbers in parentheses mean the number of patients for each category. Red color means the presence of significant coronary artery disease in accordance with ischemic heart failure criteria. ARVC = arrhythmogenic right ventricular cardiomyopathy, LV = left ventricular, EP = epicardial pattern, MDE = myocardial delayed enhancement, MW = midwall pattern, PA = patchy pattern, SE = subendocardial pattern, TR = transmural pattern.

Two radiologists (8 and 10 years of experience in cardiac imaging, respectively), who were blinded to each patient’s clinical findings, reviewed the CCTA and DECT images independently. On CCTA, coronary arteries were evaluated with percent diameter stenosis; minimal (<25%), mild (25–49%), moderate (50–69%) and severe (≥70%) [22]. Significant stenosis was defined with ≥50% diameter reduction, and when multiple lesions existed in a given artery, the artery was classified by its worst lesion. The final results were determined in consensus after assessment of interobserver agreements. On DECT, MDE was visually defined with an obviously higher intensity within the myocardium at the narrow window width and level (approximately 200 and 100 HU, respectively). The observers identified the presence of MDE and determined patients with infarction. MDE patterns were classified as follows [23]: (1) midwall–enhancement at the portion of the myocardium located between the endocardium and epicardium, (2) epicardial–enhancement of the outermost portion of the myocardium beneath the pericardium, (3) patchy–spotty or nodular enhancement, (4) subendocardial–enhancement at the innermost layer of the myocardium close to the ventricular cavity, or (5) transmural–enhancement extending from the endocardium through to the epicardium. Infarction was defined as subendocardial and transmural MDE corresponding to a perfusion territory of a coronary artery. If not the MDE pattern for infarction, the nonischemic pattern was considered which spares the subendocardium or is inconsistent with the perfusion territory of a coronary artery. Inconsistent cases were resolved in a consensus reading and observers classified the patients into groups according to MDE pattern and location (Figure 2).

Finally, the observers considered the CCTA and DECT findings together to identify possible etiologies of HF in consensus. Ischemic etiology was determined with the following criteria of prior literature [24]: patients with severe stenosis at the left main or proximal left anterior descending coronary artery; severe stenosis in two or more epicardial vessels, or infarction. For nonischemic etiologies, the tentative etiologies of HF were determined according to groups defined by findings from prior literature [25]. In addition, the observers considered ancillary findings of the myocardium, such as myocardial low attenuation <−10 HU [26] or prominent trabeculation, indicated by a thin, compacted epicardial layer and a much thicker trabecular endocardial layer [27]. The results of the combined cardiac CT were revealed to the attending HF physicians to help the initial management of newly diagnosed HFrEF patients. However, the tentative etiology determination of HF based on combined cardiac CT findings was done independently.

### 2.4. Clinical Follow-Up

Each patient was followed for 2 years after an initial diagnosis of HF. Two cardiologists independently reviewed the electronic medical records of each patient to collect all available clinical test data, including results for electrocardiography, cardiac stress tests, echocardiography, conventional coronary angiography (CAG), endomyocardial biopsy and cardiac magnetic resonance imaging (CMR) during the study period. In addition, pertinent clinical history was also recorded for medical therapy and cardiac procedures such as revascularization of coronary arteries and cardiac surgery. Finally, the final etiology of HF based on clinical follow-up was confirmed for each patient. To minimize the possibility of bias from unblinded endpoint assessment, and to increase the accuracy of diagnosis, etiology determination was done two years after combined cardiac CT evaluation. 

### 2.5. Statistical Analysis

All statistical analyses were performed using statistical software (R program, version 3.5.0.; R Foundation for Statistical Computing, Vienna, Austria). Continuous variables were presented with mean values and standard deviations. Categorical variables were presented with the numbers of patients. Inter-observer agreements for CAD evaluation, and for the detection and patterns of MDE, were analyzed with kappa statics using contingency tables. The κ values were interpreted as follows: 0.00–0.20, slight agreement; 0.21–0.40, fair agreement; 0.41–0.60, moderate agreement; 0.61–0.80 good agreement; and 0.81–1.00, excellent agreement. Concordance between tentative etiologies from cardiac CT and final etiologies from clinical decisions was assessed with the Clopper-Pearson exact binomial test with a null hypothesis probability of 0.8. There was no missing data in this study. In addition, 95% confidence intervals were calculated. 

## 3. Results

### 3.1. Baseline Characteristics

The baseline characteristics of the study participants are summarized in Table 1. Combined cardiac CT was performed successfully in all participants. The mean heart rate during cardiac CT was 74.3 ± 12.8 bpm. The effective radiation dose of CT was calculated using a cardiac-specific conversion factor: dose-length product x 0.014 mSv/(mGy·cm) [28], and the mean value was 1.22 ± 0.74 mSv for CCTA and 5.32 ± 1.85 mSv for DECT. 

### 3.2. Safety of Combined CT 

We prospectively evaluated the safety of combined CT in both inpatients and outpatients. Among 63 patients there was no radiocontrast dye allergic reaction. Regarding the prevention of CI-AKI, intravenous normal saline hydration was done for all hospitalized inpatients depending on effective circulating volume status, while oral hydration was only encouraged for outpatients. When CI-AKI was defined as an absolute increase in serum creatinine levels by ≥0.5 mg/dL, or a relative increase in serum creatinine by ≥50% from baseline is observed within 72 h after contrast exposure, only two patients (3.2%) showed CI-AKI. However, their renal function was recovered spontaneously without any further intervention. There was no contrast-related urgent hemodialysis or death during the study period.

### 3.3. Cardiac CT Findings

On CCTA, the two radiologists independently identified significant CAD with excellent inter-observer agreement (κ = 0.806) (Table 2). Afterwards, the observers determined significant CAD in 20 patients in consensus (31.7%, 20/63); 1-vessel disease in seven patients, 2-vessel disease in seven patients, and 3-vessel disease in six patients. 

On DECT, inter-observer agreement was excellent for assessment (κ = 0.806) (Table 2). Afterwards, the observers concluded that MDE was observed in 41 patients (65.1%, 41/63), with infarction in 15 patients (23.8%, 15/63) and nonischemic patterns in 26 patients (41.3%, 26/63) in a consensus reading. Twenty-two patients showed no MDE (Group I). For the 15 patients with infarction (Group II), nine patients demonstrated multifocal subendocardial and transmural MDE at multi-vessel territories, five showed subendocardial or transmural MDE at the left anterior descending coronary artery territory, and one showed transmural MDE at the right coronary artery territory. For the nonischemic patterns, seven patients showed midwall MDE at the basal septum, four showed patchy MDE at basal junctions and three showed both patterns (Group III). In addition, 10 patients showed multifocal epicardial and/or patchy MDE (Group IV), and two showed global subendocardial MDE (Group V).

### 3.4. Tentative Etiologies of HF from Cardiac CT

The main results of tentative etiologies are described in Figure 2. All 15 patients with infarction on DECT (Group II) were diagnosed as ischemic HF according to the criteria and showed significant CAD on CCTA; 1-vessel disease in four patients, 2-vessel disease in five patients, and 3-vessel disease in six patients. For these patients, infarctions were observed at the corresponding vascular territories. Additionally, three patients of Group I were regarded as ischemic HF because one patient showed 1-vessel disease with severe stenosis at the left anterior descending coronary artery and two patients showed 2-vessel disease with severe stenosis at the left anterior descending coronary artery and right coronary artery on CCTA.

**Figure 3:**
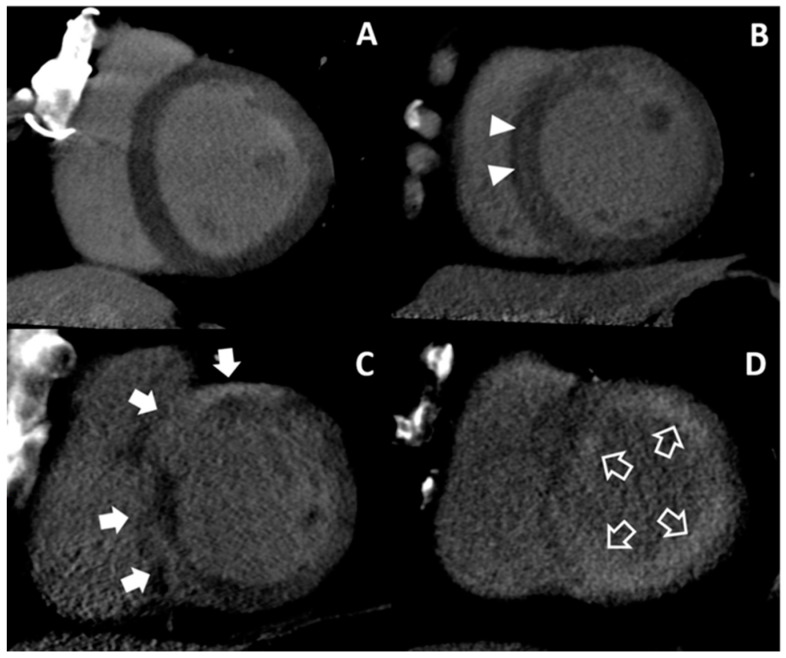
Representative cases of nonischemic heart failure. No MDE is noted on the LV (Group I) (**A**). Ill-defined midwall MDE (arrow heads) is presented in the mid LV septum (Group III) (**B**). Multifocal epicardial MDEs (arrows) are noted in the basal septum (Group IV) (**C**). Global subendocardial MDE (open arrows) is demonstrated along the LV (Group V) (**D**). LV = left ventricle, MDE = myocardial delayed enhancement.

Eighteen patients of Group I and 14 patients of Group III had dilated cardiomyopathy. One patient of Group I showed significant 1-vessel disease on CCTA but was not regarded as ischemic HF because the diseased vessel was in the left circumflex artery without perfusion defect. Other patients with tentative diagnosis of dilated cardiomyopathy did not show significant CAD on CCTA. Eight patients of Group IV were supposed as having sarcoidosis or myocarditis without significant CAD on CCTA. One patient of Group IV was diagnosed with arrhythmogenic right ventricular cardiomyopathy because of multifocal low attenuations which did not correspond to the vascular territory at both ventricles on cardiac CT. One patient of Group I and one patient of Group IV were diagnosed as isolated LV noncompaction with prominent trabeculations according to the diagnostic criteria of non-compacted-to-compacted thickness ratio >2.3 [27]. For two patients of Group V, amyloidosis was considered. Although one patient with presumed amyloidosis had significant 1-vessel disease on CCTA, ischemic HF was not considered because of moderate stenosis in the left circumflex artery. Representative cases of nonischemic HF are shown in Figure 3. 

### 3.5. Final Etiologies of HF from Clinical Decisions and Concordance Between Tentative and Final Etiologies

**Figure 4:**
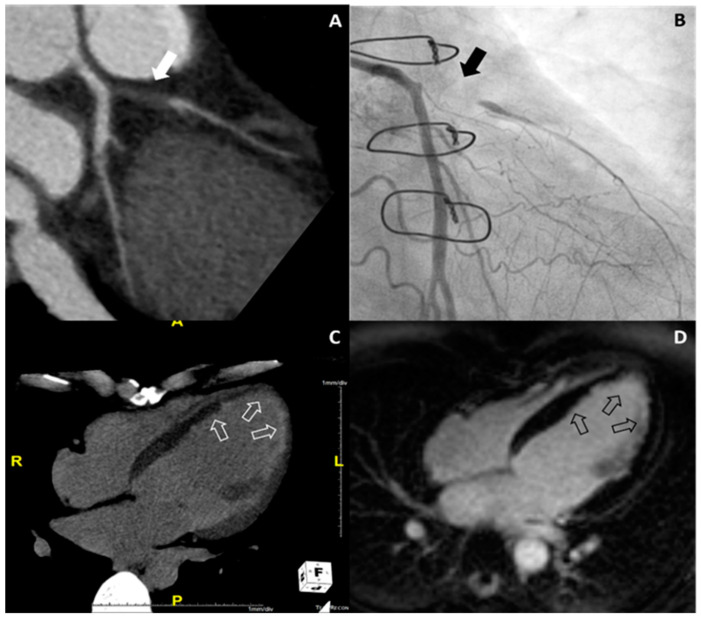
Representative case of ischemic heart failure. On coronary CT angiography (**A**), total occlusion of the proximal left anterior descending artery (white arrow) is noted, and this finding was confirmed on conventional coronary angiography (black arrow) (**B**). On delayed-enhanced dual-energy CT with the four-chamber plane (**C**), subendocardial delayed enhancement is noted on the apical left ventricle and septal wall of the mid left ventricle (white open arrows), which was confirmed on cardiac MR (black open arrows) (**D**).

Details of the results from clinical follow-up are described in the Supplemental materials. Of 18 patients with ischemic HF from cardiac CT, 16 patients underwent CAG and significant CAD was confirmed. In Group II, two patients refused to undergo CAG and were diagnosed as ischemic HF based on CMR findings with myocardial infarction. One representative case of ischemic HF is shown in Figure 4.

Among 32 patients with a tentative diagnosis of dilated cardiomyopathy, 26 patients underwent CMR. Of them, one patient of Group I was newly diagnosed as LV noncompaction with CMR [27]. For the other 25 patients, different etiologies were not suggested with CMR. In addition, different etiologies were not suggested for six patients without CMR during the two-year follow-up. All eight patients with tentative diagnosis of sarcoidosis or myocarditis underwent CMR and the diagnoses were the same as those found with CMR. These eight patients subsequently underwent cardiac biopsy. Two patients were diagnosed as cardiac sarcoidosis [29] and two patients were confirmed as myocarditis [30] according to each diagnostic criterion. The other three patients were finally regarded as unspecified cardiomyopathy and one patient as arrhythmogenic right ventricular cardiomyopathy [31]. CMR and clinical findings finally concluded in the same diagnosis for a patient with a tentative diagnosis of arrhythmogenic right ventricular cardiomyopathy [31]. Patients with a tentative diagnosis of LV non-compaction showed consistent findings on CMR as well [32]. Two patients with a tentative diagnosis of amyloidosis were pathologically confirmed with cardiac biopsy.

**Figure 5:**
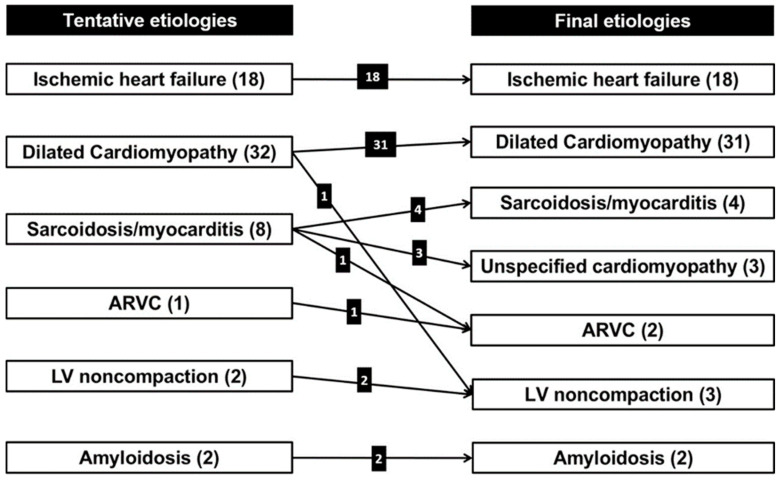
Differences between tentative etiologies from cardiac CT and final etiologies from clinical decisions. Numbers in parentheses mean the number of patients for each category. ARVC = arrhythmogenic right ventricular cardiomyopathy, LV = left ventricular.

To summarize, five patients of the 63 study participants showed different etiologies for HFrEF between cardiac CT and clinical follow-up (Figure 5). Concordance between tentative diagnoses and final diagnoses was 92.1% (95% confidence intervals of 82.7% to 96.6%). The result was significantly higher than the reference value of 0.8 (*p* = 0.017).

## 4. Discussion

The principal finding of this proof-of-concept study was that a relatively high degree of concordance was achieved between tentative etiologies from cardiac CT and final diagnoses from clinical decisions for the evaluation of underlying etiologies of HFrEF, even after exclusion of patients with a clinically presumed definite etiology of HF including history of CAD, valvular heart disease, tachyarrhythmia, chemotherapy and congenital heart disease. Combined cardiac CT, which can evaluate the coronary anatomy and myocardial fibrosis comprehensively, is a feasible, effective and safe imaging tool in the initial etiology evaluation of newly diagnosed HFrEF patients.

Once a HF diagnosis is established, the next process is determining its etiology. To do this, we need to narrow down the potential causes of newly diagnosed HF by examining the possibility of CAD [10]. Hence, cardiac CT could be recommended to exclude significant CAD in patients with low to intermediate pre-test probabilities of CAD, or those with equivocal stress tests, to minimize unnecessary invasive procedures during the HFrEF etiology work up [1,11]. In the current study, 31.7% (20/63) of the patients had newly diagnosed significant CAD on CCTA. Because most patients with longstanding ischemic cardiomyopathy usually have evidence of prior or recent myocardial infarction, nonischemic cardiomyopathy might be suggested in patients who do not show MDE [19]. However, we found five patients in Group I (22.7%, 5/22) with newly diagnosed significant CAD without infarction. Because significant CAD and nonischemic HF are not mutually exclusive, simultaneous assessment of coronary arteries and myocardial disease is needed to comprehensively evaluate HF etiologies. The presence of CAD represents a potentially treatable cause of HF while also being synergistically and independently associated with worse long-term outcomes. CAD per se would be important in the treatment of newly diagnosed HF [33].

The locations and patterns of MDE on CMR are often distinct, so a pattern-based approach for MDE might provide useful diagnostic information on the underlying myocardial disease in HF [25]. Although previous studies have steadily demonstrated that cardiac CT showed promise in MDE assessment compared to CMR, most studies focused on the evaluation of MDE itself, such as enhancement pattern and location, extent of infarction or fibrosis quantification [12,13,14,15]. Therefore, we tried to focus on the utility of cardiac CT for the evaluation of underlying etiologies in newly diagnosed HF through MDE imaging in addition to coronary artery evaluation. In the current study, we found that five patients showed discordant results between cardiac CT and clinical follow-up, and four of them belonged to Group IV with multifocal epicardial and patchy MDE. Dilated cardiomyopathy should be considered primarily for Group I (no MDE) or III (mid-wall MDE at basal septum or patchy MDE at basal junctions), whereas various less common myocardial diseases would be considered for Group IV. Because we determined a specific myocardial disease based on MDE alone for our study, the concordance between tentative etiology and final decision for the patients in Group IV might be low. Therefore, clinical features should be considered when determining a specific cause of HF in real practice. We would expect the clinical value of cardiac CT with MDE to increase with an analysis of clinical features in HF. Although we focused on coronary arteries and MDE imaging with cardiac CT in the current study, cardiac CT also provides reliable information on cardiac structure, cardiac function, cardiac venous anatomy and the pulmonary venous system, which are all considered important in HF management [34]. While cardiac CT has limitations concerning radiation exposure and iodinated contrast agents, it has several strong advantages over CMR, as whole myocardial coverage is possible with shorter times and as it is more readily available to patients. Additionally, cardiac CT can be an important alternative to CMR in patients with claustrophobia or CMR-unsafe devices.

The present study has several limitations. First, a relatively small number of patients was included in this proof of concept study. The ratio of ischemic versus nonischemic HF in the current study was different from the real world because we excluded patients with a known clinical cardiovascular history, especially those with known CAD. In addition, most patients were diagnosed as dilated cardiomyopathy (49.2%, 31/63), probably idiopathic, and ischemic HF (28.6%, 18/63), with other cardiomyopathies making up only a small portion of the study participants. This might have resulted in the high degree of concordance seen in our study. Second, not all patients underwent CAG, especially those with no significant CAD on CCTA. This finding might be due to the well-known high negative predictive value of CCTA in the evaluation of CAD. Third, the cardiac CT protocol should be modified before it can be applied to daily clinical practice. Because our scanner previously did not permit prospective ECG-gated acquisition with the dual-energy technique, we performed retrospective ECG-gated acquisition for DECT. However, prospective ECG gating reduces radiation exposure and now it is possible to do so with the dual-energy technique. Fourth, performing cardiac CT might increase iodinated contrast agent loads and radiation exposure in patients who have compelling indication for invasive coronary angiography. For those high-risk patients, risk and benefit should be fully discussed in advance.

## 5. Conclusions

To determine the upstream pathophysiology and underlying etiology of newly diagnosed HFrEF, appropriate evaluation for both coronary arteries and myocardial fibrosis is necessary. Combined cardiac CT could be a useful one-stop-imaging tool for comprehensive evaluation of underlying etiologies by making it possible to evaluate coronary anatomy and myocardial fibrosis simultaneously. Combined cardiac CT is a feasible, safe and effective imaging tool in the initial etiology evaluation for newly diagnosed HFrEF patients.

## Figures and Tables

**Table 1:** Baseline characteristics of the study population.

Age	62.6 ± 16.1
Male gender	31 (49.2%)
BMI, kg/m^2^	24.1 ± 5.0
Inpatient: Outpatient	47 (74.6%): 16 (25.4%)
NYHA class	
Class I	4 (6.3%)
Class II	40 (63.5%)
Class III	17 (27.0%)
Class IV	2 (3.2%)
Laboratory findings	
White blood cell (×10^3^/μL)	7.1 ± 2.1
Hemoglobin (g/dL)	13.5 ± 2.47
Albumin (g/dL)	3.8 ± 0.6
Cholesterol (mg/dL)	149.6 ± 36.9
Sodium (mmol/L)	140.1 ± 2.6
eGFR (mL/min/1.73 m^2^)	86.7 ± 16.8
Creatinine (mg/dL)	0.8 ± 0.2
NT-proBNP (pg/mL)	4802.4 ± 6124.1
Clinical history	
Hypertension	32 (50.8%)
Diabetes mellitus	23 (36.5%)
Dyslipidemia	8 (12.7%)
Current/ex-smoker	13 (20.6%)/8 (12.7%)
Alcohol	16 (25.4%)
Echocardiography findings	
LVEDD, mm	64.3 ± 6.7
LVEF, %	28.3 ± 8.2
Fractional shortening, %	21.8 ± 12.6

Values are *n* (%) or mean ± SD. BMI = body mass index, NYHA = New York Heart Association, eGFR = estimated glomerular filtration rate, NT-proBNP = *n*-terminal of the prohormone brain natriuretic peptide, LVEDD = left ventricular end-diastolic diameter, LVEF = left ventricular ejection fraction.

**Table 2:** Inter-observer agreement for main findings on cardiac CT.

		Observer 1	Observer 2	Kappa
CAD	No significant CAD	43	44	0.806
	1-vessel disease	6	7	
	2-vessel disease	8	6	
	3-vessel disease	6	6	
MDE	No	21	22	0.806
	Infarction	15	16	
	Non-ischemic	27	25	

CAD = coronary artery disease, MDE = myocardial delayed enhancement.

## Supplementary Materials

The following are available online at https://www.mdpi.com/2077-0383/9/7/2270/s1, Figure S1: Flow diagram of enrolled study subjects and reasons for exclusion.
