# Supplementary material for: Role of Cardiac Computed Tomography for Etiology Evaluation of Newly Diagnosed Heart Failure with Reduced Ejection Fraction
Source: J Clin Med. 2020 Jul 17;9(7):2270. doi: 10.3390/jcm9072270 (PMC7408702; doi:10.3390/jcm9072270)
Supplement: Supplementary file 1 [file jcm-09-02270-s001.pdf]

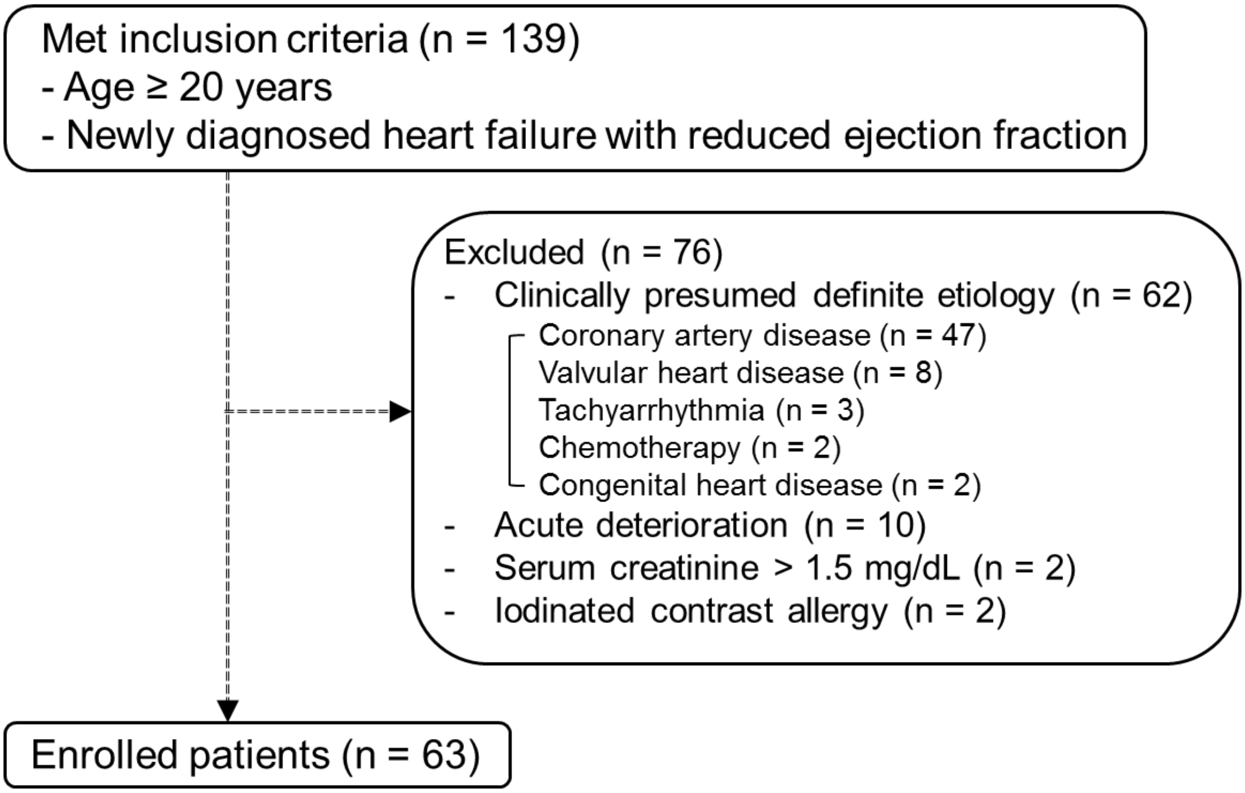

Figure S1. Flow diagram of enrolled study subjects and reasons for exclusion.

Expanded Results

Clinical follow-up of the study participants

There were 18 patients with ischemic heart failure (HF) diagnosed on cardiac CT in the current study. Of 15 patients having infarction with coronary artery disease (CAD) (Groups II), 2 patients refused to undergo conventional coronary angiography. For the patients of Group II with CAG, 1 patient with 3-vessel disease and 1 patient with 2- vessel disease on CCTA showed 2- vessel disease and 1- vessel disease on CAG, respectively. Other patients with ischemic HF showed the same results on CAG with CCTA including 3 patients of Group I. On CMR, 3 patients of Group I showed no MDE, and 15 patients of Group II showed infarction as was seen on cardiac CT.

Of 15 patients with infarction, 10 patients underwent revascularization with percutaneous coronary intervention being performed in 6 patients and coronary artery bypass graft in 4 patients after a cardiac

stress test. All 3 patients of ischemic HF without infarction on cardiac CT demonstrated no visible infarction on CMR and underwent percutaneous coronary intervention. In addition, 5 patients of ischemic HF received an implantable cardioverter defibrillator insertion within a 2-year follow-up.

Among 32 patients with a tentative diagnosis of dilated cardiomyopathy, 26 patients underwent CMR. Of them, 1 patient of Group I was newly diagnosed as LV non-compaction with CMR. For the other 25 patients, different etiologies were not suggested with CMR. In addition, different etiologies were not suggested for 6 patients without CMR during the 2-year follow-up. All 8 patients with a tentative diagnosis of sarcoidosis or myocarditis underwent CMR and the diagnoses were the same as those found with CMR. These 8 patients subsequently underwent cardiac biopsy. Two patients were diagnosed as cardiac sarcoidosis, and 2 patients were confirmed as myocarditis according to each diagnostic criteria. The other 3 patients were finally regarded as unspecified cardiomyopathy and 1 patient as arrhythmogenic right ventricular cardiomyopathy. CMR and clinical findings finally concluded in the same diagnosis for a patient with a tentative diagnosis of arrhythmogenic right ventricular cardiomyopathy. Patients with a tentative diagnosis of LV non-compaction showed consistent findings on CMR as well. Two patients with a tentative diagnosis of amyloidosis were pathologically confirmed with cardiac biopsy. Five dilated cardiomyopathy patients, 1 sarcoidosis patient, and all arrhythmogenic right ventricular cardiomyopathy patients received an implantable cardioverter defibrillator implantation. One dilated cardiomyopathy patient, 1 myocarditis patient, 2 patients with unspecified cardiomyopathy, and 1 patient with left ventricular non-compaction underwent heart transplantations.
